# Industry-sponsored economic studies in oncology *vs* studies sponsored by nonprofit organisations

**DOI:** 10.1038/sj.bjc.6601308

**Published:** 2003-10-14

**Authors:** M Hartmann, H Knoth, D Schulz, S Knoth

**Affiliations:** 1 Faculty of Medicine, Friedrich-Schiller-University of Jena, Germany; 2 Department of Statistics, Europa-Universität Viadrina, Frankfurt/Oder, Germany

**Keywords:** sponsorship, conflict of interest, oncology, health economics

## Abstract

The purpose of this analysis of health economic studies in the field of oncology was to investigate among sponsored studies whether any relationship could be established between the type of sponsorship and (1) type of economic analysis, (2) health technology assessed, (3) sensitivity analysis performed, (4) publication status, and (5) qualitative conclusions about costs. The Health Economic Evaluations Database (HEED, version 1995–2000) was searched on the basis of oncological ICD-9 codes, sponsorship, and comparative studies. This search yielded a total of 150 eligible articles. Their evaluations were prepared independently by two investigators, on the basis of specific criteria. When evaluators disagreed, a third investigator provided a deciding evaluation. There was no statistically significant relationship between the type of sponsorship and sensitivity analysis performed (*P*=0.29) or publication status (*P*=0.08). However, we found a significant relationship between the types of sponsorship and of economic analysis (*P*=0.004), the health technology assessed (*P*<0.0001), and qualitative cost assessment (*P*=0.002). Studies with industrial sponsorship were 2.56 (99% lower confidence interval (CI)=1.28) times more likely to involve cost-minimisation analyses, were 0.04 (99% higher CI=0.39) times less likely to investigate diagnostic screening methods, and were 1.86 (99% lower CI=1.21) times more likely to reach positive qualitative conclusions about costs than studies supported by nonprofit organisations. In conclusion, our results suggest that there is a greater probability that industry-sponsored economic studies in the field of oncology tend to be cost-minimisation analyses, to investigate less likely diagnostic screening methods, and to draw positive qualitative conclusions about costs, as compared to studies supported by nonprofit organisations.

There has long been discussion as to whether commercial sponsorship of clinical studies produces a conflict of interests (Davidson, 1986; Rochon *et al*, 1994; Krimsky and Rothenberg, 1998; Smith, 1998; Stelfox *et al*, 1998; Montaner *et al*, 2001; Morin *et al*, 2002). However, only two applied studies deal with this issue for health economic studies conducted in the field of oncology. Friedberg *et al* (1999) found that, in the case of new drugs developed for oncological use (including haematopoietic growth factors, antiemetics, taxanes), pharmaceutical sponsorship of economic analyses was associated with a low likelihood of reporting unfavourable results. Subsequently, however, the same authors moderated these conclusions about costs in a comparison of industry *vs* nonprofit-sponsored economic analyses of six novel drugs used in oncology (Knox *et al*, 2001). After reviewing all the available pharmacoeconomic reports, they established the differences in study reporting – but not in the types of journals in which the studies were published – between pharmaceutical company- and non-profit-sponsored studies. They concluded that these results, and in particular the observed differences in data generalisability, may have accounted in part for their previous conclusions about costs that unfavourable results were less likely to be reported from pharmaceutical-sponsored studies.

Therefore, it was the purpose of the present investigation to determine a statistically significant relationship between the type of sponsorship and (1) type of economic analysis, (2) the health technology assessed (e.g., drugs, screening, or surgery), (3) sensitivity analysis performed, (4) publication status, and (5) qualitative conclusions about costs in health economic studies conducted in the field of oncology.

## MATERIALS AND METHODS

Using the ICD-9 codes (140–165, 170–175, 179–184, 185–208, and 284), all health economic publications in the field of oncology (*n*=1381) were identified in the Health Economic Evaluations Database (HEED, Version 1995–2000). Databases covered in HEED are Medline and Embase. After eliminating redundant publications (*n*=161), letters-to-editors, review articles, editorials and methodological studies (*n*=329), a total of 891 applied studies remained. We excluded all studies (*n*=1603) not disclosing a sponsor, so as to exclude studies that were not published or did not reveal the sponsor to the editors in order to have indisputable data for our study question. The reason that we did not send letters to the studies where no sponsor was indicated to see who actually sponsored the study was that if someone has not disclosed any sponsor for a sponsored study in his publication he has done a scientific misconduct and it is unlikely that he will admit this.

**Figure 1:**
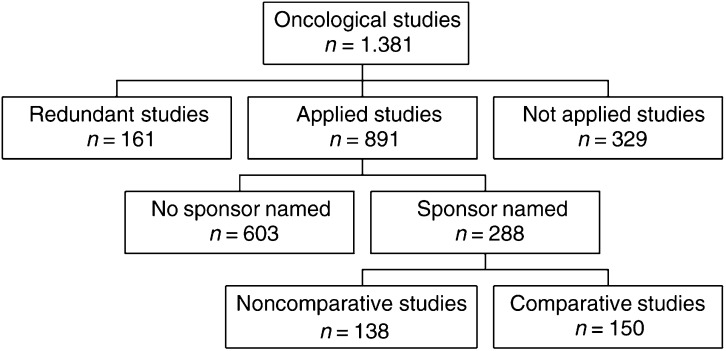
Flow diagram of the studies.

For 288 applied studies, a sponsor was named. After the exclusion of studies that did not compare alternative health technologies (cost-analysis (*n*=24), cost-consequence (*n*=98), and cost-of-illness (*n*=16) studies), a total of 150 sponsored studies involving comparisons remained (Figure 1

). They were evaluated on the basis of the criteria type sponsorship, type of economic analysis, health technology assessed, sensitivity analysis performed, publication status, and qualitative conclusions about costs.

An investigation was compared to have been industry-sponsored or to have been sponsored by nonprofit organisations if, in the publication, it was disclosed that all or part of the support provided originated from industry, respectively, from nonprofit organisations. For assessing the type of economic analysis used, distinctions were made among *cost-effectiveness analysis* (measures benefits of different medical treatments having a common health outcome (e.g., life years saved) and computes a cost-effectiveness measure ratio for comparison), *cost-benefit analysis* (measures costs and benefits of a medical treatment in financial units and computes a net monetary gain/loss or a cost–benefit ratio), *cost-utility analysis* (measures benefits of different medical treatments in utility-weighted life years (quality-adjusted life years, QALYs) and computes a cost–utility ratio for comparison), and *cost-minimisation analyses* (finds the least costly medical treatment among those shown or assumed to be of equal medical benefit).

Studies were also differentiated into categories for health technology assessed: pharmacokinetic monitoring, supportive care, medical devices, diagnostic, drugs, screening, and surgery. Publication status as a marker for the publication rated studies as published in peer-reviewed or non-peer-reviewed journals. Since even within the peer-reviewed literature, the quality of publications is highly variable, a sensitivity analysis (one-way or multiple-way) was chosen from the checklist for this subject, developed by Drummond (Drummond and Jefferson, 1996) as an additional marker for the study quality. Sensitivity analysis (one-way or multiple-way) was chosen, because it is a process through which the robustness of an economic model is assessed by examining the changes in results of the analysis when key variables are varied over a specified range. Therefore, a yes/no decision was made as to whether a sensitivity analysis had been performed. Qualitative conclusions about costs were evaluated as positive, negative or neutral. Conclusions about costs were assumed to be positive if there was a statement in the publication that the health technology assessed reduced costs, was cost effective, cost beneficial, or cost utilitarian, and negative if the technology assessed had higher cost, and was not cost effective, cost beneficial, or cost utilitarian. When there was neither a positive nor a negative statement about the outcome of cost assessment, it was assumed to be neutral for the purpose of the present analyses. All evaluations were made independently by two investigators (MH and HK). If the investigators disagreed over the evaluation of any article, a third investigator (DS) made a deciding evaluation.

Relationships between the type of sponsorship and type of economic evaluation, health technology assessed, sensitivity analysis performed, publication status, and qualitative conclusions about costs were analysed using Fisher's exact test (the modifications by Metha for tables larger than 2 × 2) to provide a two-sided probability (*P*<0.05, against the null hypothesis of no relationship was considered significant).

In a second analysis, large tables (analysis type, health technology, and conclusions about costs) were recombined to provide 2 × 2 tables. That is, for dealing with the relative risk (industry *vs* nonprofit) for ‘cost-minimisation’ analyses, all other types were collected in one category called ‘other’. The reason behind the collapsing into 2 × 2 tables was not the (two-sided) *P*-value itself, but the one-sided *P*-value, because Fisher's exact test is inherently two-sided (at least for tables larger than 2 × 2); furthermore by collapsing into 2 × 2 tables, we could construct confidence intervals (CIs) for the relative risk for ‘cost minimisation,’ which is not possible for larger tables. Hence, by using these 2 × 2 tables, one-sided 99% CIs for the relative risks were calculated by computing *P*-values. A (small) one-sided *P*-value allows to conclude that one sponsoring type provides significant larger (or smaller) numbers of, for example, positive outcomes. The higher confidence level of 99% was chosen so as to insure that simultaneous computing of two (ore more) intervals provides reasonable small overall error probabilities and does not lead to high error probabilities. No formal adjustment was made for multiple comparisons and, therefore, the 99% CIs have descriptive interpretation rather than being used for hypothesis testing. All computations were executed by SAS 8.2 Software (SAS Institute Inc., Cary, NC, USA).

## RESULTS

The sponsor of 71% (106 out of 150) of the studies evaluated was a nonprofit organisation; the other 44 studies (29%) were supported by an industrial sponsor in whole or part. Of the 150 studies, 63% were cost-effectiveness analyses, 22% cost-minimisation analyses, 12% cost-utility analyses, and 3% involved cost–benefit analyses. The treatments assessed were drugs in 41% of the 150 studies, screening procedures in 29%, diagnostic methods in 14%, surgery in 12%, care in 2% (3), medical devices in 2%, and pharmacokinetic monitoring in 1%. In 48% of the 150 studies, there was no sensitivity analysis, and nearly all (89%) were published in peer-reviewed journals. The two primary evaluators agreed on the classification in 82% of the cases, and the third investigator was required to determine the classification of the other 18%.

The type of sponsorship was not significantly associated with sensitivity analysis or publication status (*P*=0.29, 0.08). However, there was a demonstrable relationship between the type of sponsorship and type of economic analysis (*P*=0.004), health technology assessed (*P*<0.0001), and qualitative conclusions about costs (*P*=0.002) (Table 1

**Table 1:** Study set characteristics and conclusions

**Characteristic**	**Sponsorship industry (*n*=44)**	**Sponsorship nonprofit (*n*=106)**	***P*-value**
*Analysis type*			
Cost minimisation	17	16	
Cost effectiveness	19	76	0.004
Cost utility	6	12	
Cost–benefit	2	2	
			
*Technology assessed*			
Pharmaceuticals	36	25	
Surgery	3	15	
Screening	1	42	
Diagnostics	3	18	<0.0001
Devices	1	2	
Supportive care	0	3	
Pharmacokinetic monitoring	0	1	
			
*Publication status*			
Non-peer reviewed	8	8	0.08
Peer reviewed	36	98	
			
*Sensitivity tested*			
Yes	26	52	0.29
No	18	54	
			
*Qualitative conclusions about costs*			
Positive	27	35	
Negative	7	16	0.002
Neutral	10	55	

). Compared to studies sponsored by nonprofit organisations, industry-sponsored studies were 2.56 (lower 99% CI=1.28, therefore >1.00) times more likely to be cost-minimisation analyses and 0.60 (higher 99% CI=0.92, or <1.00) times less likely to be cost-effectiveness analyses. They were also 3.45 (lower 99% CI=2.24) times more likely to investigate drugs and 0.04 (higher 99% CI=0.39) times less likely to investigate screening. In comparison with the studies sponsored by nonprofit-making organisations, they were 1.86 (lower 99% CI=1.21) times more likely to reach positive qualitative conclusions about costs.

## DISCUSSION

This investigation encountered several interesting relationships between the type of sponsorship and the outcome of health economic studies conducted in the field of oncology, despite some limitations. We emphasise that we did not investigate individual studies for possible selecting bias that may have arisen from limiting studies considered to those in which information on financial support was provided. In addition, we did not stratify any subgroup based on the quality of the studies.

Our results show that, irrespective of the study sponsor, three times as many studies with positive rather than negative conclusions about costs (62 *vs* 23 studies) were published. This outcome can be attributed to the fact that in health economic research, as in other areas of medicine (Callahan *et al*, 1998), there is a preference for publishing studies with positive results (publication bias). Although this practice is considered a scientific misconduct, withholding the publication of unfavourable results is not uncommon (Bodenheimer, 2000). Furthermore, industry-sponsored studies were 1.9 times more likely to have positive qualitative conclusions about costs than studies sponsored by nonprofit organisations. This relationship can be attributed to a sponsorship bias. Industry-sponsored clinical studies more frequently compare novel treatments against a placebo than against drugs that are known to be effective (Djulbegovic *et al*, 2000). Therefore, it can be assumed that companies may simply avoid conducting head-to-head economic trials, particularly when they are unlikely to reveal the superiority of a new treatment or drug.

In comparison with studies sponsored by nonprofit organisations, industry-sponsored studies were 1.9 times more likely to be cost-minimisation analyses, and 2.5 times less likely to be cost-effectiveness analyses. The reason for these relationships becomes apparent when the definition of cost-minimisation analysis is considered. Such analyses involve comparisons of equieffective alternatives on the basis of net costs, with the aim of determining the less costly option. If it can be demonstrated in a cost-minimisation analysis that, with identical clinical outcome, the sponsor's drug reduces costs of treatment compared to a competitor's drug, the sponsor can readily expand market share. Drug companies can use such successful trials to market their products. This factor could also explain the most important demonstrated association between industry sponsorship and positive qualitative cost assessment, if cost-minimisation analysis is inherently more likely to lead to a positive assessment.

Sensitivity analysis is useful for determining the quality of investigations in health economics. It is a process through which the robustness of an economic study is assessed by examining the changes in results of the analysis when key variables are varied over a specified range. However, in nearly half (72/150=46%) of the present studies, no sensitivity analysis was conducted, irrespective of the type of study sponsor. On the basis of this criterion alone, almost half of all health economic studies conducted in the field of oncology have an evidence level below 3b, according to the classification system of the Oxford Centre for Evidence-based Medicine (2002). They thus have the same value for health economic analysis as case series (evidence level 4) or expert opinions without critical appraisal (evidence level 5) have for therapeutic aspects.

In comparison to studies sponsored by nonprofit organisations, the industry-sponsored studies were 3.45 times more likely to involve drugs and 25 (1/0.04) times less likely to involve evaluation of diagnostic screening methods. For industry, in contrast with nonprofit organisations, the economic evaluation of screening methods is usually of limited financial interest. However, in view of the fact that a medical treatment exhibits a reduction of its marginal utility with increasing exploitation, the economic evaluation of screening procedures is of particular interest. Only by means of health economics assessment is it possible to provide the preparatory groundwork for the decision on who should be screened, how often, and at what cost.

In order to counter the risk that the type of sponsorship might influence health economic analyses, it is necessary to improve the quality of such studies. Economic analyses should: (1) be based on clinically acceptable costs or alternatives, (2) conduct a systematic review of the evidence, and (3) include a multiway sensitivity analysis (simultaneous variation of several key variables to test the robustness of the result), in order to achieve an evidence level comparable to that of randomised controlled clinical trials (RCTs) [12]. Furthermore, full disclosure of all financial interests involved in health economic studies should be provided routinely.

As we could demonstrate, the potential for bias may exist on multiple levels. Therefore, establishing checks and balances for academic–industry partnership, as it is proposed for clinical studies (Chopra, 2003), may help to mitigate the potential for bias in health economics.
